# Molecular detection of fluoroquinolone-resistance in multi-drug resistant tuberculosis in Cambodia suggests low association with XDR phenotypes

**DOI:** 10.1186/1471-2334-11-255

**Published:** 2011-09-28

**Authors:** Corinne Surcouf, Seiha Heng, Catherine Pierre-Audigier, Véronique Cadet-Daniel, Amine Namouchi, Alan Murray, Brigitte Gicquel, Bertrand Guillard

**Affiliations:** 1 Mycobacteriology Laboratory, Institut Pasteur du Cambodge, Phnom Penh, Cambodia; 2 Unité de Génétique Mycobactérienne, Institut Pasteur, Paris, France

## Abstract

**Background:**

Drug susceptibility testing (DST) remains an important concern for implementing treatment of MDR tuberculosis patients. Implementation of molecular tests for drug resistance identification would facilitate DST particularly in developing countries where culturing is difficult to perform. We have characterized multidrug resistant strains in Cambodia using MDTDR*sl *tests, drug target sequencing and phenotypic tests.

**Methods:**

A total of 65 non-MDR and 101 MDR TB isolates collected between May 2007 and June 2009 were tested for resistance to fluoroquinolones and aminoglycosides/cyclic peptides using the GenoType^® ^MTBDR*sl *assay and gene sequencing. Rifampicin resistance (RMP-R) was tested using gene sequencing and genotyping was assessed by spoligotyping.

**Results:**

A total of 95 of the 101 MDR strains were confirmed to be RMP-R by *rpoB *gene sequencing. Fourteen of the 101 MDR isolates (14%) carried a *gyrA *mutation associated with fluoroquinolone-resistance (FQ-R) (detected by the MTBDR*sl *assay and sequencing) compared with only 1 (1.5%) of the 65 non-MDR strains. Only 1 (1%) of the MDR isolates was found to be XDR TB. The MDR group contained a higher proportion of Beijing or Beijing like strains (58%) than the non MDR group (28%). This percentage is higher in MDR FQ-R strains (71%).

**Conclusions:**

The new GenoType^® ^MTBDR*sl *assay combined with molecular tests to detect RMP-R and isoniazid resistance (INH-R) represents a valuable tool for the detection of XDR TB. In Cambodia there is a low rate of XDR amongst MDR TB including MDR FQ-R TB. This suggests a low association between FQ-R and XDR TB. Strain spoligotyping confirms Beijing strains to be more prone to accumulate antibiotic resistance.

## Background

The World Health Organization (WHO) estimated that there were 0.5 million cases of multi-drug-resistant (MDR) tuberculosis (TB) in 2007. Only 8.5% of the estimated global total of smear-positive cases of MDR-TB were notified. By the end of 2008, 55 countries and territories had reported at least one case of extensively drug-resistant TB (XDR-TB) which are defined as MDR strains that are also resistant to a fluoroquinolone (FQ) and at least one second-line injectable agent (amikacin (AM), kanamycin (KM) and/or capreomycin (CM)) [1].

The WHO underlines the absolute necessity to rapidly scale up the diagnosis and effective disease management of MDR-TB and highlights the problem of access to drug susceptibility testing (DST) for second-line drugs (SLD). Indeed, only nine of the 22 high burden countries (HBCs), who account for 80% of incident TB cases, had access to second-line DST [2].

In 2007, Cambodia with a population of 14.4 million, ranked 21^st ^among the HBCs for TB. The incidence of all forms of TB in this country in 2007 was estimated at 495/100,000 population with a mortality rate at 89/100,000 population [1]. The directly observed therapy strategy (DOTS) has covered 100% of the TB cases for more than 10 years, with 93% treatment success and the number of sputum-smear positive MDR-TB cases (in 2007) estimated to be 94 cases [1]. The incidence of MDR-TB appears to have remained relatively low. In 2001, a study of drug resistance carried out by the National Tuberculosis Control Program of Cambodia reported the absence of MDR among new cases and a rate of 0.4% of MDR-TB among all TB cases [3]. Nevertheless, a study undertaken between March 2003 and February 2005 found a rate of 5.1% of MDR-TB among HIV co-infected tuberculosis patients in Phnom Penh [4]. In 2007, the HIV prevalence among TB patients was 7.8% and 46% of TB relapses were MDR (data not published). It is essential that TB remains under active surveillance in Cambodia and MDR-TB diagnosis must be further developed. However, until 2011, no laboratory in Cambodia had second-line DST capability. The aim of this retrospective study was to evaluate the prevalence of XDR, among MDR strains. Rifampicin-resistance (RMP-R) was confirmed using *rpoB *gene sequencing. The GenoType^® ^MTBDR*sl *assay was used for detection of resistance to FQ, and aminoglycosides/cyclic peptides (AG/CP) using *gyrA*, and *rrs *target genes. Spoligotyping was used to determine the rate of strain transmission among the MDR population.

## Methods

### Strains

MDR strains were collected at the Institut Pasteur of Cambodia between May 2007 and June 2009 from all patients diagnosed with MDR TB after drug susceptibility testing. Non MDR strains were selected randomly during the same period. In total, 65 non MDR and 101 MDR *M. tuberculosis *strains were isolated in the Mycobacteriology Laboratory at the Institut Pasteur du Cambodge (IPC, Phnom Penh). DST for first-line TB drugs was performed using the BD MGIT reading manual method before May 2009 and by the MGIT Bactec 960 method after this date. DNA was extracted from the isolates and used for sequencing, MTBDR*sl *assay and spoligotyping.

### GenoType^® ^MTBDR*sl *assay

The GenoType^® ^MTBDR*sl *assay provided by BIOCENTRIC, Bandol, France, was used to determine resistance to FQ, and AM/CM. Tests were performed according to the manufacturer's instructions.

### DNA sequencing of the *rpoB, gyrA, rrs *genes

Genes were amplified by PCR and then sequenced using a BigDyeTerminator kit and an ABI Prism model 3100 DNA sequencer. Primer sequences are given in Table 1.

**Table 1:** Primers used for detection of antibiotic resistance

Antibiotic	Gene	Primers	Product size (bp)
**Rifampicin**	*rpoB*	TR1 (5'-TACGGTCGGCGAGCTGATCC-3')	411
		TR2 (5'-TACGGCGTTTCGATGAACC-3')	
**Fluoroquinolones**	*gyrA*	gyrA-F (5'-GATGACAGACACGACGTTGC-3')	398
		gyrA-R (5'-GGGCTTCGGTGTACCTCAT-3')	
**Kanamycin/Amikacin**	*rrs*	Rrs F (5'-AAACCTCTTTCACCATCGAC-3')	1329
		Rrs R (5'-GTATCCATTGATGCTCGC-3').	

bp: base pair

### Spoligotyping

Spoligotyping was performed as previously described [5].

## Results

A total of 101 MDR strains and 65 non MDR strains previously characterized by conventional DST were investigated in this study. Eighty-six MDR strains were also resistant to streptomycin (SM). Results are presented in tables 2 and 3.

**Table 2:** Results of phenotypic testing, sequencing, MTBDR*sl *and spoligotyping

MDR strains							
Number of strains	DST Phenotype	Molecular testing					Spoligotype
		**RMP**	**FQ**		**KM/AM**		

		***rpoB***	***gyrA***	**MTBDR*sl***	**rrs (1401, 1402, 1484)**	**MTBDR*sl***	

1	INH, RMP, SM	S531L	A90V	r	a1401g	r	EAI2-NTB

1	INH, RMP, SM	D516V	A90V mixed wt	r	wt	s	Beijing

1	INH, RMP, SM	S531L	D94A	r	nd	s	Beijing

2	INH, RMP, SM	S531L	D94A	r	wt	s	Beijing

1	INH, RMP, SM	H526Y	D94A	r	wt	s	Beijing

1	INH, RMP, SM	S531L	D94A	r	wt	s	Beijing

1	INH, RMP, SM	S531L	D94A	r	wt	s	Beijing

1	INH, RMP, SM	L533P	D94A mixed wt	r	wt	s	Beijing

1	INH, RMP, SM	D516V	D94G	r	wt	s	Beijing

1	INH, RMP, SM	H526D	D94G	r	wt	s	Beijing

1	INH, RMP, SM	S531L	D94G	r	wt	s	EAI1_SOM

1	INH, RMP, SM	S531L	D94G	r	wt	s	U

1	INH, RMP, SM	S531L	D94G mixed wt	r	wt	s	U

1	INH, RMP	S531L	wt	s	wt	s	Beijing

1	INH, RMP	S531L	wt	s	wt	s	Beijing

1	INH, RMP	S531L	wt	s	wt	s	Beijing

1	INH, RMP, SM	H526N L533P	wt	s	wt	s	Beijing

1	INH, RMP, SM	H526R	wt	s	wt	s	Beijing

1	INH, RMP, SM	R529Q mixed wt	wt	s	wt	s	Beijing

1	INH, RMP, SM	D516G S531L	wt	s	wt	s	Beijing

1	INH, RMP, SM	D516V	wt	s	wt	s	Beijing

1	INH, RMP, SM	D516V	wt	s	wt	s	Beijing

1	INH, RMP, SM	D516V	wt	s	wt	s	Beijing

1	INH, RMP, SM	D516V	wt	s	wt	s	Beijing

1	INH, RMP, SM	D516V	wt	s	wt	s	Beijing

1	INH, RMP, SM	D516V mixed wt	wt	s	wt	s	Beijing

1	INH, RMP, SM	D516V mixed wt; S531L	wt	s	wt	s	Beijing

1	INH, RMP, SM	insertion ttc 514; A532V	wt	s	nd	s	Beijing

1	INH, RMP, SM	S531L	wt	s	wt	s	Beijing

1	INH, RMP, SM	S531L	wt	s	wt	s	Beijing

1	INH, RMP, SM	S531L	wt	s	wt	s	Beijing

1	INH, RMP, SM	S531L	wt	s	wt	s	Beijing

1	INH, RMP, SM	S531L	wt	s	wt	s	Beijing

1	INH, RMP, SM	S531L	wt	s	wt	s	Beijing

1	INH, RMP, SM	S531L	wt	s	wt	s	Beijing

1	INH, RMP, SM	S531L	wt	s	wt	s	Beijing

2	INH, RMP, SM	S531L	wt	s	wt	s	Beijing

1	INH, RMP, SM	wt	wt	s	wt	s	Beijing

1	INH, RMP, SM	S512N T525T H526P L527Q	wt	s	wt	s	Beijing

1	INH, RMP, SM	H526D	wt	s	wt	s	Beijing

1	INH, RMP, SM	H526Y	wt	s	wt	s	Beijing

1	INH, RMP, SM	H526Y	wt	s	wt	s	Beijing

2	INH, RMP, SM	H526N L533P	wt	s	wt	s	Beijing

1	INH, RMP, SM	H526R	wt	s	wt	s	Beijing

1	INH, RMP, SM	H526R	wt	s	wt	s	Beijing

1	INH, RMP, SM	L533P	wt	s	wt	s	Beijing

1	INH, RMP, SM	D516V mixed wt; S531L	wt	s	wt	s	Beijing

1	INH, RMP, SM	insertion ttc 514	wt	s	wt	s	Beijing

1	INH, RMP, SM	insertion ttc 514	wt	s	wt	s	Beijing

2	INH, RMP, SM	S531L	wt	s	wt	s	Beijing

1	INH, RMP, SM	S531L	wt	s	wt	s	Beijing

1	INH, RMP, SM	S531L	wt	s	wt	s	Beijing

2	INH, RMP, SM	S531L	wt	s	wt	s	Beijing

1	INH, RMP, SM	S531L	wt	s	wt	s	Beijing

1	INH, RMP, SM	L524W T525P del 526	wt	s	wt	s	Beijing

1	INH, RMP, SM	wt	wt	s	wt	s	Beijing

1	INH, RMP, SM	S531L	wt	s	wt	s	Beijing-like

1	INH, RMP, SM	wt	wt	s	wt	s	Beijing-like

1	INH, RMP	del 518	wt	s	wt	s	EAI1_SOM

1	INH, RMP	S531L	wt	s	wt	s	EAI1_SOM

1	INH, RMP	wt	wt	s	nd	s	EAI1_SOM

1	INH, RMP, SM	S531L	wt	s	wt	s	EAI1_SOM

1	INH, RMP, SM	H526D	wt	s	wt	s	EAI1_SOM

1	INH, RMP, SM	S531L	wt	s	nd	s	EAI1_SOM-EAI2

1	INH, RMP, SM	S531L	wt	s	wt	s	EAI4_VNM

1	INH, RMP, SM	H526D	wt	s	wt	s	EAI5

2	INH, RMP, SM	D516Q Q517N del 518	wt	s	wt	s	EAI5

1	INH, RMP, SM	S531L	wt	s	nd	s	EAI5

1	INH, RMP	L533P	wt	s	wt	s	EAI5

1	INH, RMP	S531L	wt	s	wt	s	EAI5

1	INH, RMP	L533P	wt	s	wt	s	U

1	INH, RMP	D516V	wt	s	wt	s	U

1	INH, RMP, SM	D516V mixed wt	wt	s	wt	s	U

1	INH, RMP, SM	H526D	wt	s	wt	s	U

2	INH, RMP, SM	H526D	wt	s	wt	s	U

1	INH, RMP, SM	S531L	wt	s	wt	s	U

1	INH, RMP, SM	S531L	wt	s	wt	s	U

1	INH, RMP	H526D mixed wt	wt	s	wt	s	Unknown

1	INH, RMP	S531L	wt	s	wt	s	Unknown

1	INH, RMP	S531L	wt	s	wt	s	Unknown

1	INH, RMP	wt	wt	s	wt	s	Unknown

1	INH, RMP	wt	wt	s	wt	s	Unknown

1	INH, RMP, SM	H526Y	wt	s	wt	s	Unknown

1	INH, RMP, SM	H526D R529Q	wt	s	wt	s	Unknown

2	INH, RMP, SM	S531L	wt	s	wt	s	Unknown

1	INH, RMP, SM	S531L	wt	s	wt	s	Unknown

1	INH, RMP, SM	H526Y	wt	s	wt	s	Unknown

1	INH, RMP, SM	D516V	wt	s	wt	s	Unknown

1	INH, RMP, SM	D516V	wt	s	nd	s	Unknown

1	INH, RMP, SM	S531L	wt	s	wt	s	Unknown

1	INH, RMP, SM	S531L	wt	s	wt	s	Unknown

1	INH, RMP, SM	S531L	wt	s	wt	s	Unknown

1	INH, RMP, SM	D516G	wt	s	nd	s	ZERO

INH isoniazid, RMP rifampicin, SM streptomycin

AM amikacin, FQ fluoroquinolones, KM kanamycin

wt wild type, s susceptible, r resistant, nd not determined

**Table 3:** Results of phenotypic testing, sequencing and spoligotyping

non MDR strains				
**Number of strains**	**DST Phenotype**	**Sequencing**		**Spoligotype**

		***rpoB***	**gyrA**	

1	INH, SM	wt	wt	Beijing

3	SM	wt	wt	Beijing

13	no resistance	wt	wt	Beijing

1	no resistance	wt	wt	Beijing-like

1	SM	wt	wt	EAI_SOM

1	no resistance	wt	V55V	EAI_SOM

1	INH	wt	wt	EAI1_SOM

4	no resistance	wt	wt	EAI1_SOM

1	no resistance	wt	wt	EAI1_SOM

1	no resistance	wt	wt	EAI2_MANILLA

1	no resistance	wt	wt	EAI2_MANILLA

1	INH	Q513K	wt	EAI5

1	INH	wt	wt	EAI5

1	INH	wt	wt	EAI5

1	no resistance	wt	wt	EAI5

2	no resistance	wt	wt	EAI5

4	no resistance	wt	wt	EAI5

1	no resistance	wt	wt	EAI5

1	no resistance	wt	wt	EAI5

1	no resistance	wt	wt	EAI5

1	no resistance	wt	wt	H3

1	INH, SM	wt	wt	U

2	no resistance	wt	wt	U

1	INH, SM	wt	D94A	Unknown

1	INH	wt	wt	Unknown

1	INH	wt	wt	Unknown

1	RMP	H526Y	wt	Unknown

1	RMP	wt	wt	Unknown

1	no resistance	wt	wt	Unknown

1	no resistance	wt	wt	Unknown

1	no resistance	wt	wt	Unknown

1	no resistance	wt	wt	Unknown

1	no resistance	wt	wt	Unknown

1	no resistance	wt	wt	Unknown

1	no resistance	wt	wt	Unknown

1	no resistance	wt	wt	Unknown

1	no resistance	wt	wt	Unknown

1	no resistance	wt	wt	Unknown

1	no resistance	wt	wt	Unknown

1	no resistance	wt	wt	Unknown

1	no resistance	wt	wt	Unknown

1	no resistance	wt	wt	Unknown

1	no resistance	wt	wt	Unknown

INH isoniazid, RMP rifampicin, SM streptomycin, wt wild type

### RMP-R

A total of 95 out of 101 (94%) MDR strains with a RMP-R phenotype carried a mutation known to confer resistance in the core region of the *rpoB *genes (table 2). The codons most frequently involved were codon 516 (18/101 = 18%), codon 526 (22/101 = 22%) and codon 531 (47/101 = 47%). All of these mutations can be detected by the GenoType^® ^MTBDR*plus *assay. Eighty-three strains showed a single mutation (codons involved: 516, 526, 529, 531 and 533) and 3 had a repetition of codon 514, which is associated with the mutation A532V. Ten strains displayed several mutations (or a deletion for one strain) with at least one known to be associated with RMP resistance. One strain showed a previously un-published mutation (L524W) associated with deletion of codon 526. In six strains that were phenotypically RMP-R, the sequence of the core region of *rpoB *was identical to the wild type. Because most RMP-R strains are known to be isoniazid resistant (INH-R), molecular INH resistance was not checked to confirm phenotypic isoniazid resistance and RMP-R strains were considered as MDR strains.

### FQ-R

Mutations known to be associated to FQ-R were found in 14 out of 101 MDR strains (14%) and 1 out of the 65 non MDR strains (1.5%). The MTBDR*sl *assay detected all 14 FQ-R mutations that were confirmed by *gyr*A sequencing. The codons involved were 94 (gac D94A gcc, n = 7; gac D94G ggc, n = 5) and 90 (gcg A90V gtg, n = 2) (table 2). In 3 of these 14 strains, a superposition of nucleotides was observed suggesting that they were a mixture of wild type and mutant populations. All the strains were shown to carry a mutation at codon 21 [6] and 95 [7] of the *gyrA *gene. This polymorphism is not associated with resistance. The FQ-R group contained a higher proportion of Beijing strains identified by spoligotyping (10/14, 71%) than the FQ-S group (47/87, 54%).

### *rrs *gene

The *rrs *gene was sequenced in 95 strains to identify resistance to AM, KM and/or CM. For 6 strains, sequencing data was not interpretable because of bacteriological contamination. The MTBDR*sl *assay detected only one strain with a1401 g mutation in the *rrs *gene. No other strain had a mutation at codon 1401, 1402 or 1484, which are all known to be associated with resistance to AM, KM and CM. Among the MDR strains, all except one of the 14 FQ-R strains were found to be AM-S. The AM-R strain was also FQ-R, thus, this single XDR strain represents 1% of the MDR population.

### Strain diversity

Spoligotyping results showed that the majority of MDR strains belonged to the Beijing family (57/101, 56%) or were Beijing like (2/101, 2%). This percentage is higher in MDR FQ-R strains (10/14, 71%). This confirms that Beijing strains are more prone to accumulate antibiotic resistances. The other families were: U (10/101), EA15 (6/101), EAI1_SOM (6/101), EAI1_SOM-EA12 (1/101), EA12-NTB (1/101), EA14-VNM (1/101), ZERO (1/101) and 16 Unknown profiles. In contrast, only 17 Beijing and 1 Beijing like out of 65 non MDR strains (28%) were identified as such. The other families were: U (3/65), EA15 (13/65), EAI_SOM (2/65), EAI1_SOM (6/65), EA12_MANILLA (2/65), H3 (1/65), and 20 Unknown profiles. MDR strain comparisons using spoligotype profiles and target gene mutations allowed us to differentiate 74/101 strains indicating that they are not epidemiologically related. The other MDR strains were grouped as follows: 27 strains were grouped into 10 pairs (6 pairs of Beijing and 4 non Beijing) and one group of three Beijing and one group of four Beijing strains.

## Discussion

This study analyzed resistance of Cambodian MDR-TB strains to major anti-tuberculosis drugs.

Unpublished data showing that 46% of TB relapses in Cambodia are MDR is consistent with previous reports from the Central African Republic [8], Japan [9], and South Africa [10].

RMP-R was confirmed with *rpoB *core region sequencing in 94% of MDR strains. This agrees with other studies showing that some RMP-R strains carry a wild type *rpoB *core region sequence [11]. *rpoB *core region mutations were in the hot spot positions associated with RMP-R strains [12,13]. We observed a high number of MDR strains carrying several *rpoB *mutations. This may suggest a stepwise process of additive mutations (as described for FQ-R) that could generate higher levels of resistance to RMP [14-16].

To evaluate the number of XDR strains among MDR strains we identified mutations in *gyrA *and *rrs *genes which are associated with FQ resistance and AG/CM resistance respectively. Our study found that 14% of clinical MDR-TB isolates were FQ resistant. This rate is lower than that found in other studies of MDR-TB in Southern Asia: Taiwan 22.2% [17] or 42.8% using phenotypic DST; Shanghai 25.1% [18] and the Philippines 17% [19] or 51.4% [20]. However, these figures are much higher than those found on other continents (4.1% in MDR-TB in the United States and Canada [21] and 4.3% in MDR-TB in Russia [22]). Only one of the MDR FQ-R strains was AM-R. Therefore, only 1% of MDR strains are identified as XDR strains. This shows that identification of only *gyrA *FQ-R mutations is not a strong indication of an XDR phenotype.

Previous studies [23,24] showed a high level of concordance between MTBDR*sl *and phenotypic tests: I.E. 90.2% and 75.6% respectively. In this study, we had no access to phenotypic tests for FQ. MTBDR*sl *and sequencing data of *gyrA *are 100% concordant and these techniques identified 14% FQ-R in MDR strains. This FQ-R rate may have been underestimated because of the unavailability of FQ phenotypic testing in Cambodia. FQ resistance is most often associated with *gyrA *mutations [25,26] and more particularly mutations at codons 94 and 90. This was the case in our study. The percentage of FQ-R *M. tuberculosis *clinical isolates with *gyrA *mutations have been reported to be between 70% and 90% [23]. Because we have no access to FQ-R phenotypic tests, our analysis may have missed a number of cases. However, as reported before [23,24], in the absence of phenotypic tests, molecular tests will detect more than 75% of FQ-R cases. Mutations other than those affecting *gyrA *and other mechanisms could result in FQ-R, including: decreased cell-wall permeability to drug, efflux pumps, drug sequestration or perhaps even drug inactivation [12,26]. In a small number of cases, FQ-R could be associated with *gyrB *mutations and a probable efflux mechanism [27,28].

FQ has become an essential part of treatment regimens for MDR tuberculosis [25,26]. Due to their efficacy and safety, the new generation of FQ's is even being evaluated as a first-line medication for tuberculosis [27-29]. Unfortunately, the extensive use of FQ has increased spontaneous acquisition of mutations associated with FQ-R. It has been suggested that routine FQ-R testing in locations where resistant strains are endemic may be clinically useful by showing a significant correlation between development of FQ-R and first-line *M. tuberculosis *drug resistance [30]. However, extensive use of FQ would likely increase MDR tuberculosis treatment failure. As in other developing countries, problems arise from the uncontrolled use of antibiotics. However, it is interesting to note that only 1 out of 14 MDR FQ-R strains is XDR, thus showing that detection of FQ-R strains cannot be used as a single marker for the detection of XDR cases. Additional tests are required to identify XDR cases, among them molecular tests like MTBDR*sl *that include the detection of mutations in *gyrA *associated to FQ-R and *rrs *associated with AM, KM and CM resistance.

Work by other researchers has demonstrated that the MTBDR*sl *assay detected 86.7% (39/45) and 100% (5/5) of phenotypically AM and CM resistant TB strains [23,24]. The mutation a1401 g was the most prevalent. The XDR isolate in our study was confirmed to carry a1401 g mutation in the *rrs *gene. This result suggests a low rate of XDR amongst MDR TB in Cambodia (1%) as compared to countries like India and Taiwan that have reported rates of 8-15% and 10% respectively [31,32]. This favorable situation could be linked to the implementation of the DOTS strategy in 1980 by the national TB control program in Cambodia [3].

Spoligotyping confirmed previous observations that the Beijing family is prevalent in Asia. The higher proportion of Beijing spoligotypes in the MDR group (58%) compared with the non MDR group (28%) is consistent with previous studies demonstrating that strains of the Beijing genotype more readily acquire resistance mutations than non-Beijing strains [33-35]. This percentage is increased in the MDR FQ-R group (71%). Although most of the spoligotypes had the Beijing profile, no major MDR outbreak by this family has been reported in Cambodia. Further studies with new markers are warranted to provide a more accurate picture of the epidemiology of the Beijing strains in Cambodia.

## Conclusions

A molecular study of 101 MDR *M. tuberculosis *strains in Cambodia identified only 1 (1%) XDR strain although a high number of the MDR cases showed FQ-R (14%). The new GenoType^® ^MTBDR*sl *assay represents a reliable tool for the detection of FQ and AG/CAP resistance and in combination with molecular tests for the detection of RMP-R and INH-R, XDR TB can potentially be identified within 1 or 2 days. These tests could provide valuable information to facilitate the management of patient therapy and the prevention of transmission.

## Authors' contributions

CS participated in the planning of the study, acquisition of samples and demographic data; SH participated in culture and isolation of mycobacteria, molecular assays; CPA participated in data analysis and drafting of the manuscript; VCD, AM and AN participated in molecular assays, data analysis and drafting of manuscript; AM participated in general supervision of the study and critical revision of the manuscript; BG and BG the conception of the study, general supervision of the study, critical revision of manuscript. All authors read and approved the final manuscript.

## Pre-publication history

The pre-publication history for this paper can be accessed here:

http://www.biomedcentral.com/1471-2334/11/255/prepub
